# HEALTH INFLUENCES OF UNIQUE AND INTERSECTING IDENTITIES: AGE, SEXUAL ORIENTATION AND RACE

**DOI:** 10.1093/geroni/igac059.3039

**Published:** 2022-12-20

**Authors:** Laura Bernstein, Julie Patrick

**Affiliations:** West Virginia University, Morgantown, West Virginia, United States; West Virginia University, Morgantown, West Virginia, United States

## Abstract

Lesbian, gay, and bisexual older adults are an understudied group with limited research using nationally representative samples. Although there are known physical health disparities based on age, sexual orientation, and race, the effects of intersecting identities have not been fully examined. We were interested in how these identities individually influence health and whether they interact to effect physical health. Using the 2020 Behavioral Risk Factor Surveillance System (M age = 54.30, SD = 17.52), we examined whether sexual orientation was related to the number of poor physical health days experienced and whether that relation was moderated by age and race. The overall model was significant (F (5, 223356) = 511.86, p < .001, R2 = .0113). Significant direct effects of sexual orientation and race emerged, but these were qualified by a significant sexual orientation by race interaction (b = -.09) A significant main effect emerged for age (b = 1.08) but the sexual orientation by age interaction failed to reach significance. There was a significant three-way interaction between age, race, and sexual orientation (F (2, 223356) = 6.34, p < .01). Investigation into the means found that multiracial adults tend to report more poor health days than White, Black, or Hispanic adults, except for bisexual older and younger adults. These results highlight the importance of examining intersecting identities when trying to understand health disparities across the lifespan. Understanding how these identities interact helps to identify specific barriers these groups face and ultimately improve health equity, specifically in an aging population.

